# Comparison of choroidal thickness in eyes of diabetic patients with eyes of healthy individuals using optical coherence tomography in a tertiary care hospital

**DOI:** 10.12669/pjms.38.1.4443

**Published:** 2022

**Authors:** Hafsa Hassan, Alyscia Cheema, Muhammad Ali Tahir, Hina Nasreen Nawaz

**Affiliations:** 1 Hafsa Hassan, MBBS, FCPS. Jinnah Post Graduate Medical Centre, Karachi, Pakistan; 2 Alyscia Cheema, MBBS, FCPS, FRCS. Professor of Ophthalmology Jinnah Post Graduate Medical Centre, Karachi, Pakistan; 3 Muhammad Ali Tahir, MBBS, FCPS (Ophthalmology), FCPS (Vitreoretina) Consultant Retinal Surgeon Jinnah Post Graduate Medical Centre, Karachi, Pakistan; 4 Hina Nasreen Nawaz, MBBS, MRCS. TRMO, Jinnah Post Graduate Medical Centre, Karachi, Pakistan

**Keywords:** Choroidal thickness, Swept source optical coherence tomography, Diabetic retinopathy

## Abstract

**Objectives:**

To compare the choroidal thickness in eyes of diabetic patients with eyes of age matched controls using optical coherence tomography in a tertiary care hospital.

**Methods:**

This Cross sectional study was conducted at the Department of Ophthalmology, Jinnah Postgraduate Medical Centre Karachi, for six months from13^th^January 2020 to13^th^July 2020. The study group comprised of 44 patients with 88 eyes. Patients who fulfill the inclusion criteria that is age ranging from 35 to 80years, either gender, known case of diabetes mellitus and having any type of diabetic retinopathy (HbA1c >7), non-diabetic healthy individuals (HbA1c < 7) and those giving informed consent were included in the study. However, patients having active ocular infections, history of myocardial infarction, stroke, uveitis, any ocular surgery, lasers, intravitreal injections, poor fundus view and not giving consent were excluded. A pre-designed proforma was filled. A baseline ocular examination was performed and choroidal thickness was assessed from retinal pigment epithelium to choroid sclera junction in diabetic and healthy participants of the study group using high resolution Swept source OCT (DRI-OCT-2 Triton; Topcon).

**Results:**

The average age of the patients was 39.41±15.95 years. According to our study mean central subfoveal choroidal thickness in diabetic eyes was 268.5 ± 66.22 (95% CI 240 – 297) and in non-diabetic healthy participants it was 339.3 ± 71.49 (95% CI 308 – 369) with a p-value of 0.001. However, average choroidal thickness was 261.8 ± 61.93 (95% CI 235 – 288) and 336.0 ± 74.35 (95% CI 304 – 367) in diabetic and non-diabetic healthy population with a p-value of 0.001. Choroidal thickness comparison between gender in diabetic and non-diabetic population also showed similar trend.

**Conclusion:**

In this study, mean central choroidal thickness as well as average choroidal thickness was significantly reduced in eyes having diabetic retinopathy as compared to participants with non-diabetic healthy eyes. These findings indicate that changes in choroid may be a probable route in the pathogenesis of diabetic retinopathy.

## INTRODUCTION

The rise of diabetic population in the world has led to a number of problems. Diabetic retinopathy (DR) damages vision by causing abnormalities in retinal microvasculature and capillaries.^1^ The disease is described by development of micro aneurysms, perfusion abnormalities of capillaries, and ischemic retina, eventually heading to neo vessels formation and macular edema, which can disturb visual function.^2^,^3^ Clinically significant macular edema is edema or hard exudates within 500 micrometers of the centre of macula, retinal edema one disc diameter or larger, any part of which is within one disc diameter of center of macula. The outer third of the retina gets oxygen and nutrients from choroid. The three vascular layers of choroid are chorio capillaries layer, Sattler layer and Haller layer.^2^,^4^ Since there are many diseases of posterior segment which also involve choroid any assessment of morphological features and its vasculature in different chorioretinal diseases may be of clinical significance. Hemodynamic abnormalities, Changes in retinal vessels integrity, break down of blood retina barrier mark the development of diabetic eye disease.^5^ Clinicians and researchers believe that in addition to retinal changes choroidal vasculopathy might also play some role in the development of diabetic retinopathy.

Numerous choroidal malfunctions including blockades of the choriocapillaries, disintegration of vessels, aneurysms of vessels of choroid, and choroidal neovascularization have been testified in diabetic eyes.^6^ Good-quality with higher-resolution cross-sectional macula imaging is possible with Spectral-domain optical coherence tomography (OCT). Choroidal thickness is different in diabetic and non-diabetic eyes as per previous studies. Adhi et al^2^ observed alteration in morphology of choroid while comparing mild to moderate NPDR patients to normal population and showed that mean choroidal thickness was 276.4±13.4 micrometers in normal eyes while 211.6±17um in diabetic eyes. The mid of choroid is attenuated in case of diabetic retinopathy. Whereas, Lee et al^5^ showed that the mean central choroid was 229.1±16.8µm in normal eye compared to 219.7± 30.4 µm in diabetic eye. Similarly, Gerendas et al^7^ also concluded that choroidal thickness was decreased in patients with diabetes if DME was present. Since prevalence of diabetes is increasing it is imperative to prevent its ophthalmological complications for individual to live a normal life. We can prevent irreversible blindness due to diabetes by looking at choroidal thickness beforehand and patients can be counseled and treated before irreversible blindness occur. We are therefore comparing the choroidal thickness of diabetic and non-diabetic eyes using swept source OCT.

## METHODS

The study was conducted at Department of Ophthalmology, Jinnah Post-Graduate Medical Centre, Karachi from 13^th^ January 2020 to 13^th^ July 2020. Sample size calculation was done by WHO sample size calculator using the following assumptions.^8^ Confidence Interval (1-α) = 95%. Margin of error (d) 0.03 Taking choroidal thickness 259.1±13.1um. Sample size of 88 eyes of 44 patients was calculated. Out of which 44 eyes were of diabetic patients having diabetic retinopathy (HbA1c > 7) and 44 eyes were of non-diabetic healthy individuals age matched control group (HbA1c < 7). Approval of the study was done by institutional review board and ethical committee of Jinnah Post Graduate Medical Centre (Ref: No.F.2-81/2020=GENL/S374/PJMC, Dated: 03-02-2020). Patients having either gender, age between 35 to 80 years, any type of diabetic retinopathy, duration of diabetes (at least greater than one year), non-diabetic healthy individuals and those who gave consent for inclusion in the study and for OCT were included in the study. However patients having active ocular infection, uveitis, uncontrolled hypertension, pregnancy, any ocular surgery, lasers or intravitreal injections and those who did not give consent were excluded from the study. A pre-designed proforma was filled. A baseline ocular examination was performed and choroidal thickness was assessed using swept source OCT (SS-OCT) (DRI-OCT-2 Triton; Topcon). It was measured from retinal pigment epithelium to choroid scleral junction. Choroidal thickness was measured in central subfoveal region, 500um temporal to central region and 500um nasal to central sub foveal region. Central subfoveal and average thickness of three regions were compared between diabetic and non-diabetic healthy individuals of the study group. SPSS version 21 was used for data entry and analysis. Mean (SD) was computed for Age, duration of diabetes mellitus and choroidal thickness. Frequencies and percentages were calculated for categorical variables like gender, diabetics and non- diabetics. Effect modifiers such as age, was assessed through stratification. Post stratification, Dependent/paired T test was applied. P-value ≤0.05 was considered significant.

## RESULTS

Mean age of patients was 39.41±15.95 years. Average duration of diabetes was 10.64 ± 2.7 years. Out of 88 eyes of 44 patients 32 eyes (36.6%) were of female patients and 56 eyes (63.64%) were of male patients. Mean central choroidal thickness in patients having diabetic retinopathy was 268.5 ± 66.22 (95% CI 240 – 297). However, it was 339.3 ± 71.49 (95% CI 308 – 369) in normal healthy subjects hence was significantly reduced in comparison to the normal healthy subjects with a p-value of 0.001. Average choroidal thickness was calculated by taking mean of central, nasal and temporal thickness readings. Average choroidal thickness in patients having diabetic retinopathy was 261.8 ± 61.93 (95% CI 235 – 288) in comparison to 336.0 ± 74.35 (95% CI 304 – 367) in normal healthy subjects with a p-value of 0.001. Similarly, nasal and temporal thicknesses were also reduced significantly in diabetic population of the group in comparison to normal healthy population as shown in Table-I. Same trend was seen when stratification was done according to gender. Significant difference in central and average thicknesses was seen in male gender in diabetic and non-diabetic study population of group (Table-II). However in case of female gender although the difference was observed but it was not significant as shown in (Table-III). Similarly, nasal and temporal thicknesses were also reduced significantly in diabetic population of the group in comparison to normal healthy population as shown in Table-I. Same trend was seen when stratification was done according to gender. Significant difference in central and average thicknesses was seen in male gender in Diabetic and non diabetic study population of the group. (Table-II) however in case of female gender although the difference was observed but it was not significant as shown in (Table-III).

**Table-I:** Mean comparison of the Choroidal thickness in eyes of diabetic patients with eyes of healthy individuals using optical coherence tomography (OCT) (n= 44 patients with 88 eyes).

Choroidal Thickness	Diabetic (n=22)		Non diabetic (n=22)		p-value

	Mean ± S.D	(95% C.I)	Mean ± S.D	95% C.I	
Nasal	253.5 ± 62.23	(227 – 280)	330.4 ± 74.54	(298 – 362)	0.001
Central	268.5 ± 66.22	(240 – 297)	339.3 ± 71.49	(308 – 369)	0.001
Temporal	263.5 ± 61.00	(237 – 289)	388.7 ± 79.01	(304 – 372)	0.001
Average choroidal thickness	261.8 ± 61.93	(235 – 288)	336.0 ± 74.35	(304 – 367)	0.001

For Male Cases (n= 28 patients with 56 eyes).

**Table-II:** Mean Comparison of the Choroidal thickness in eyes of diabetic patients with eyes of healthy individuals using optical coherence tomography (OCT).

Choroidal Thickness (Male)	Diabetic (n=12)		Non diabetic (n=16)		p-value

	Mean ± S.D	(95% C.I)	Mean ± S.D	(95% C.I)	
Nasal	236.5 ± 61.48	(201 – 272)	333.3 ± 84.95	(290 – 375)	0.003
Central	253.6 ± 69.40	(213 – 293)	345.0 ± 80.38	(304 – 385)	0.004
Temporal	255.4 ± 63.22	(218 – 291)	343.4 ± 89.51	(298 – 388)	0.007
Average choroidal thickness	248.5 ± 63.95	(211 – 285)	340.58 ± 84.27	(298 – 382)	0.001

For Female Cases (n= 16 patients).

**Table-III:** Mean Comparison of the Choroidal thickness in eyes of diabetic patients with eyes of healthy individuals using optical coherence tomography (OCT).

Choroidal Thickness (Female)	Diabetic (n=10)		Non diabetic (n=6)		p-value

	Mean ± S.D	(95% C.I)	Mean ± S.D	(95% C.I)
Nasal	273.9 ± 59.73	(236 – 311)	322.8 ± 39.81	(290 – 355)	0.098
Central	286.4 ± 60.73	(248 – 324)	323.9 ± 41.18	(290 – 357)	0.205
Temporal	273.1 ± 60.05	(235 – 311)	324.6 ± 43.30	(289 – 360)	0.089
Average choroidal thickness	277.8 ± 58.56	(240 – 314)	323.8 ± 40.88	(290 – 357)	0.115

No significant difference were observed p>0.05.

**Fig.1:**
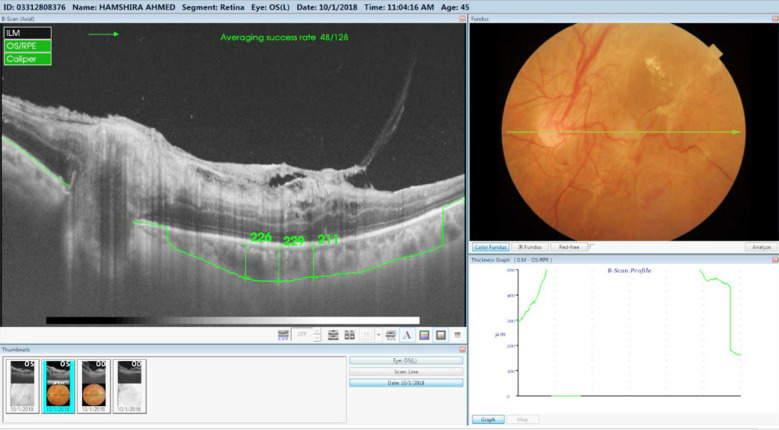
OCT image of a diabetic patient having PDR with DME illustrating choroidal thickness.

**Fig.2:**
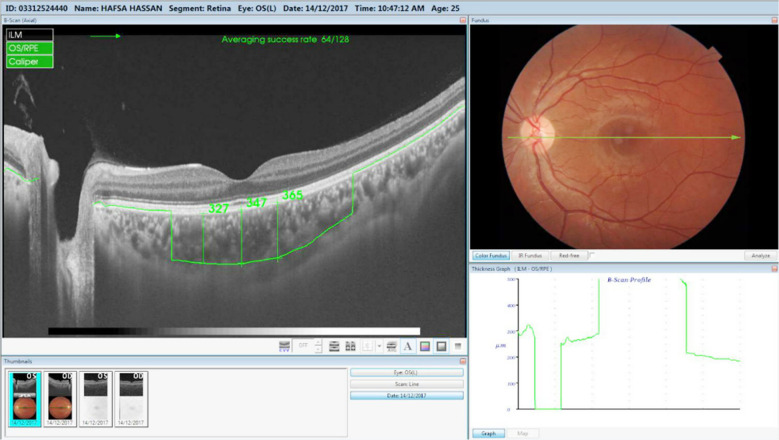
OCT image of a non diabetic healthy individual in our study group.

## DISCUSSION

Choroid having normal structure and function is imperative for the functioning of retina. Unusual blood volume of choroid or reduced blood flow can cause malfunction of photoreceptors and their death as well.^9^

Number of studies was conducted previously investigating the effect of choroidal vessels in the development of diabetic retinopathy. It was speculated that almost similar changes take place in retinal and choroidal vessels and growth factors released by diabetic retina and choroid were also similar. It was assumed that affected choroidal vascular system in diabetic patients may be associated in the development of diabetic retinopathy.^10^ SD-OCT and so does swept source OCT has helped in evaluation of cross sectional structure of choroid.^11^ and choroid scleral interface.^12^ Studies evaluating the blood flow in sub foveal choroidal region using Doppler flowmetry reveal decline in volume and blood flow in patients suffering from moderate to severe non proliferative diabetic retinopathy (NPDR) and proliferative diabetic retinopathy (PDR).^13^ Reduction in blood flow was more marked in PDR.^14^

To understand Diabetic eye disease in a better way it is important to have knowledge of choroidal damage. It is only recently that appropriate visualization of choroid has been possible using OCT in spite of difficulties like posterior location and existence of retinal pigment epithelium.^15^,^16^

In this study the average age of the patients was 39.41±15.95 years. 63.64% were male patients and 36.36% were female. In El Ghonemy et al study^17^ the mean age of the control group and the mean age of the diabetic patients were 52.88±12.9 and 56.95±8.3 years. In Wang et al study^18^ among them,58.28% patients were female, the average age was 64.5 ± 7.8 years, the average duration of diabetes was 8.9 ± 7.1 years. The difference in average age in our study group may be attributed to the patients who are coming from low socio economic status, poor control of diabetes and compliance to the treatment and follow up are also not good. Low percentage of females in our study group in comparison to Wang et al^18^ may be because of cultural values and females may not be reporting enough in comparison to males.

In our study mean central sub foveal choroidal thickness in normal healthy subjects was 339.3 ± 71.49um (CI 95% 308– 369) where as In El Ghonemy et al study^17^ mean subfoveal CT was 354.52±96.51μm in the control group 269.4±64.3μm in the mild NPDR subgroup, 240.7±67μm in the moderate NPDR subgroup, 227.7±47.9μm in the severe NPDR subgroup, 220.3±58.1μm in the PDR subgroup, and 224.4±55.3μm in the DME group. In our study mean central subfoveal choroidal thickness in eyes having diabetic retinopathy was 268.5 ± 66.22 (CI 95% 240 – 297). This value is very close to El Ghonemy et al mild and moderate NPDR sub groups.^17^

In Wang et al study^18^ the average CT was 189.2±72.6 μm for all the participants, 187.6 ± 72.5 μm for patients without DR, and 195.4 ± 72.9 μm for DR patients (P = 0.115). However, in our study average choroidal thickness in non-diabetic healthy sub group was 336.0 ± 74.35(CI 95% 304 – 367) and in diabetic sub group it was 261.8 ± 61.93 (CI 95% 235 – 288) with a p-value of 0.001. In Wang et al study among measurements in nine sub regions, the CT showed a trend toward higher values in DR patients, but only CT in outer nasal region (P = 0.015) and outer inferior region (P = 0.029) achieved statistical significance. In contrary to Wang et al study average choroidal and central subfoveal choroidal thicknesses in our study were not only higher in both diabetic and non-diabetic sub groups which may be attributed to different OCT machine used and different geographic location of people but the difference in choroidal thicknesses with in our study group were also significant with p value of 0.001. Mean central choroid was significantly low in diabetic eye as compared patients with non-diabetic eyes in this study. Whereas, Lee et al^5^ showed that the mean central choroid was 229.1± 16.8 µm in normal eye compared to 219.7± 30.4 µm in diabetic eye. Similarly, Gerendas et al^7^ also reported that choroidal thickness was reduced in patients with diabetes if DME was present. Studies done previously show that choroidal thickness varies in diabetic and non- diabetic eyes. Adhi et al^2^ showed that choroidal morphological features are altered in patients with moderate to severe diabetic retinopathy with mean chordial thickness of 276.4± 13.4 µm in normal eye while 211.6±17.0µm in diabetic eyes. Studies done previously show different results on association of choroidal thickness with severity of DR. Many authors concluded that choroidal thickness in DR patients is reduced. For example, Lains et al.^19^ demonstrated that CT in their proliferative DR group was thinner compared with controls. Horváth et al.^20^ and Ambiya et al^21^ revealed that decreasing CT correlated with the severity of DR. However, other studies reported thickening of choroid or no change with presence of DR. For example, Tavares et al.^22^ reported a thickening of choroid in diabetic patients without DR. The population-based Beijing Eye Study found that the DM was independently associated with a thicker choroid while the DR was not related to the choroidal thickness.^23^ DM may act as an independent factor leading to choroid thickening and subsequent DR progression may lead to the reduction of CT, which may appear as a thicker choroid at the initial stage of DR and thinning with DR progression. The choroid provides the outer layer of retina and the retinal pigment epithelium with oxygen and nutrients. Hence CT may give an idea about how much active retina and choroid are metabolically. The mechanism of CT alterations in DR remains unclear. Diabetic choroidopathy may result in RPE dysfunction, and affect vascular permeability.^24^ The increased choriocapillaries permeability leads to choroidal thickening. Second, the over expression of cytokines activated by inflammation, oxidative stress, angiogenesis in early DR may contribute to the thickening of the choroidal layer, such as monocyte chemotactic protein-1, platelet-derived growth factor, VEGF, insulin-like growth factor 1, pigment epithelium-derived factor, and cxc motif chemokine ligand.^25^ It was reported that these cytokines were significantly associated with choroidal thickening.^26^,^27^ Third, the choroid accounts for 85% of ocular blood flow, autonomic nervous system was considered to be important for autoregulation of choroidal blood flow. In mild NPDR, sympathetic innervation increases choroidal circulation as a result of which choroidal thickness is increased. Savage et al. reported that the pulsatile ocular blood flow increased in DR eyes compared with controls using a computerized pneumotonometer. However, hypoxia plays a dominant role with the DR progression to late stage.^28^

Thinning of the choroid suggests a decrease in blood flow, and thus thinning of the choroid may be associated with hypoxia in the retinal tissue. However, further experimental studies are needed to determine whether choroidal thinning was primary or secondary to retinal ischemia.^28^

### Limitation of the study:

It was that average and central choroidal thicknesses among diabetic population were not compared according to the severity of Diabetic retinopathy like in sub groups of mild NPDR, moderate NPDR, severe NPDR, PDR and in advanced diabetic eye diseases to had an idea of decrease in choroidal thickness with increase in severity of diabetic retinopathy and severity of ischemia. More studies with greater sample size are required to find out the association between severity of diabetic retinopathy and degree of ischemia with reduction in choroidal thickness.

## CONCLUSION

In this study mean central choroidal thickness was significantly low in diabetic eyes in comparison to patients with non-diabetic eyes. These findings lead to a point of view that change in choroidal thickness may be a route in the development of DR. More studies with greater sample size are required to find out the mechanism behind our findings.

### Authors’ Contributions:

**HH:** Conceived the study and manage data collection**.**

**AC:** Study was done under her supervision.

**MAT:** Contributed in acquisition of data, critical review, and is responsible for integrity of the study, final approval of manuscript.

**HNN:** Contributed in study design and drafting the article.
